# Dietary Copper on the Onset of Puberty in Rats: Possible Mechanism

**DOI:** 10.3390/nu17223534

**Published:** 2025-11-12

**Authors:** Rui Sun, Zhongshen Wang, Cheng Li, Meng Li, Wenyan Yang, Lianyu Yang

**Affiliations:** 1 College of Animal Science and Technology, Jilin Agricultural University, Changchun 130118, China; sr1399919804@163.com (R.S.); 15585538818@163.com (Z.W.); licheng15754365617@163.com (C.L.); 15500270655@163.com (M.L.); 2 Key Laboratory of Animal Production, Product Quality and Security, Ministry of Education, Changchun 130118, China; 3 Jilin Provincial Key Laboratory of Animal Nutrition and Feed Science, Changchun 130118, China

**Keywords:** HPOA axis, KISS-1/GPR54, PKC, GABA

## Abstract

**Background/Objectives**: Copper is an essential trace element for physiological processes related to reproduction, but its impact on the hypothalamic–pituitary–ovarian (HPOA) axis and its specific mechanism remain unclear. **Methods**: In vivo study: 21-day-old female Sprague Dawley (SD) rats were randomly assigned to five groups (*n* = 10 per group), with all groups fed a basal diet and supplemented with CuSO_4_·5H_2_O to achieve copper ion concentrations of 0, 15, 30, 45, or 60 mg/kg in the diet. During the second phase of proestrus, blood samples, hypothalamic tissues, pituitary tissues, and ovarian tissues were collected. In vitro study: Primary mixed hypothalamic neurons were isolated and cultured from fetal SD rats on embryonic day 17. After identification by NSE immunofluorescence staining, six copper ion concentration groups (0, 15.6, 31.2, 46.8, 62.4, and 78 μmol/L) were established. The optimal copper concentration for cell viability and GnRH secretion was screened using CCK-8 assay (Sangon, Shanghai, China) and ELISA (Mlbio, Shanghai, China). On this basis, the cells were treated with different concentrations of PKC agonist (PMA) and PKC inhibitor (chelerythrine). Cell viability was evaluated by CCK-8 assay, the expression level of PKC was detected by Western blot, and the optimal concentration with no obvious toxicity was selected for subsequent mechanism research. **Results**: Dietary copper dose-dependently regulated rat puberty onset; the 45 mg/kg copper group had the earliest onset, and showed significantly increased levels of reproduction-related hormones (GnRH, FSH, LH, E_2_) in serum and HPOA axis. Hypothalamic transcriptomics revealed significantly enriched GnRH signaling pathways and GABAergic synaptic pathways. Mechanistically, this copper dose upregulated hypothalamic KISS-1, GPR54, and PKC (mRNA/protein), and downregulated GABA/GABA-R. Adding 46.8 μmol/L copper (as Cu^2+^, equivalent to optimal in vivo level) could activate the KISS-1/GPR54-GnRH system in hypothalamic neurons; regulating PKC activity could synchronously affect the expression of KISS-1, GPR54, GnRH, and GABA/GABA-R, with additional copper enhancing this effect in vitro experiments. **Conclusions**: This study demonstrates for the first time that dietary copper at 45 mg/kg promotes puberty onset in SD rats. The mechanism involves activation of the hypothalamic PKC pathway, which inhibits GABAergic neurotransmission while activating the KISS-1/GPR54-GnRH system, thereby enhancing HPOA axis activity and gonadotropin secretion.

## 1. Introduction

Delayed or abnormal puberty has emerged as a pressing global public health concern, affecting approximately 5–10% of adolescents worldwide, with rates of precocious puberty in certain populations—specifically 0.67% among females in coastal China—raising particular alarm. These pubertal abnormalities not only increase the risk of reproductive disorders such as polycystic ovary syndrome and infertility but also trigger psychological challenges including anxiety and depression during this critical developmental period [1,2,3]. Puberty is driven by activation of the hypothalamic–pituitary–ovarian (HPOA) axis, wherein hypothalamic Gonadotropin-Releasing Hormone (GnRH) stimulates pituitary release of Follicle-Stimulating Hormone (FSH) and Luteinizing Hormone (LH), and these hormones in turn promote gonadal hormone synthesis and sexual maturation [4,5,6,7]. The timing of pubertal onset is co-regulated by genetic, environmental, and nutritional factors, with nutrition serving as a critical “metabolic sensor” that signals reproductive readiness [8,9]. Malnutrition, including micronutrient deficiency, increases the risk of delayed puberty, while adequate nutrition facilitates timely HPOA axis activation [10,11].

Copper is an essential micronutrient involved in neuroendocrine regulation, steroidogenesis, and antioxidant defense, all of which are integral processes to the onset of puberty [12,13,14,15]. Clinical evidence links disruptions in copper homeostasis to abnormal pubertal timing: excessive accumulation, as in Wilson’s disease, is associated with precocious puberty [16,17], whereas congenital deficiency, as in Menkes disease, often leads to delayed puberty and hypogonadism [18,19]. Existing studies mainly describe copper’s reproductive effects and toxicity at a macroscopic level, with dosage schemes largely based on standard adult or non-pregnant animal needs. Since puberty initiation and ovulation may demand higher copper availability, the precise mechanisms by which copper acts as a metabolic signal to activate the reproductive axis remain poorly understood, limiting targeted nutritional strategies and precise stage-specific regulation.

The stable function of GnRH neurons, the core regulatory unit of the HPOA axis, is essential for puberty onset. While abnormal copper levels (deficiency or excess) can disrupt GnRH synthesis and pulsatile secretion [20,21], the underlying mechanisms remain poorly defined. GnRH pulsatile release is regulated by a complex network involving peptide signaling, neurotransmitter interactions, and ion channel dynamics. Central to this network is the Kisspeptin-1/G Protein-Coupled Receptor 54 (KISS-1/GPR54) system: kisspeptin, encoded by KISS-1, binds to GPR54 on GnRH neurons to trigger pulsatile GnRH secretion and can sense metabolic signals such as leptin and insulin during puberty [22,23,24,25], a mechanism that aligns with copper’s potential role as a metabolic sensor. At the neurotransmitter level, glutamatergic excitatory and Gamma-Aminobutyric Acid (GABA) inhibitory signals, balanced via Na^+^-K^+^-2Cl^−^ Cotransporter 1 (NKCC1)/K^+^-Cl^−^ Cotransporter 2 (KCC2) ion channels, synergistically regulate GnRH neuronal activity [26,27]. Notably, copper ions can modulate GABA inhibition by blocking extrasynaptic δ-GABAA receptors [28], and this GABA signaling is further regulated by intracellular molecules including Protein Kinase C (PKC), Calcium/calmodulin-dependent protein kinaseII (CaMKII), and Cyclic Adenosine Monophosphate (cAMP) through phosphorylation-dependent mechanisms [29,30,31]. However, whether and how copper influences pubertal timing through these HPOA axis regulatory pathways remains to be elucidated.

We hypothesized that copper could influence the onset of puberty through the integrated regulation of neurotransmitter networks and peptide signaling pathways. Therefore, the aim of this experimental study was to evaluate the effects of dietary copper on age at first estrus and parameters related to the HPOA axis, identify copper-sensitive pathways, and elucidate the underlying mechanisms.

## 2. Materials and Methods

This study was conducted in two phases. The in vivo phase aimed to identify the optimal dietary copper concentration that accelerates puberty onset in rats, while the in vitro phase was designed to investigate the molecular mechanisms underlying copper’s effects on hypothalamic neurons.

### 2.1. Animal Care and Experimental Design

Fifty 21-day-old female SD rats (body weight: 44.24 ± 2.95 g) were purchased from Liaoning Changsheng Biotechnology Co., Ltd. (Shenyang, China). All rats were randomly divided into five groups (Groups A, B, C, D, and E) using the random number generation function in Microsoft Excel. Each group contained five replicates, with two rats per replicate, and the experimental unit was the individual rat. There was no significant difference in the initial body weight among the groups after grouping. To control experimental bias, a double-blind design was adopted throughout the experiment—both the personnel responsible for the daily feeding and management of the rats and those conducting subsequent sample detection and analysis were blinded. All animals were housed in the Key Laboratory of Animal Nutrition and Feed Science, Jilin Agricultural University (Jilin Province). The housing conditions were maintained as follows: they were kept in standard polypropylene cages (size: 47.5 × 35 × 20 cm) with 2 rats per cage—ensuring adequate space as recommended for juvenile rodents—alongside a relative humidity of 50%, a temperature of 22 ± 1 °C, and a 12 h light/12 h dark cycle. Daily ventilation and disinfection were also conducted. During a 5-day adaptation period, all rats were fed the AIN-93G basal diet (copper content: 6.5 ± 0.17 mg/kg), whose formulation was described in our previous study [32]. After the adaptation period, Group A (control group) continued to receive the basal diet, while Groups B, C, D, and E were fed the basal diet supplemented with 15, 30, 45, and 60 mg/kg CuSO_4_·5H_2_O (Sigma, St. Louis, MO, USA), respectively, based on the previous research foundation of our laboratory and relevant literature references [33,34]. Food and water were provided ad libitum throughout the experiment.

### 2.2. Detection of Estrous Stage and Sampling & Preservation of Blood/Tissues

Vaginal opening in rats was observed starting at approximately 33 days of age. After vaginal opening, vaginal exfoliated cells were collected daily for smear examination. The collected vaginal exfoliated cells were stained using hematoxylin solution: cells were fixed on slides, immersed in hematoxylin stain for 10 min to stain cell nuclei, and then rinsed with water to remove excess stain for clear microscopic observation. The stages of the estrous cycle (proestrus, estrus, metestrus, and diestrus) were accurately determined by observing the proportional changes in epithelial cells, keratinized cells, and leukocytes under a microscope, and the time of the first estrus was recorded. After recording the first estrus, daily observations of vaginal exfoliated cell smears were continued. The second proestrus was identified when a significant increase in the number of epithelial cells and a minimal presence of leukocytes were observed again, which matched the cellular proportional characteristics of proestrus. During this stage, all rats received a subcutaneous injection of buprenorphine (0.05 mg/kg body weight) 30 min before blood collection to alleviate procedural pain. Blood samples were then collected via venipuncture, with the operation performed by a well-trained technician to ensure rapidity and accuracy. Serum was separated by centrifugation and stored at −20 °C. The rats were subsequently euthanized (the rats were placed in a carbon dioxide anesthesia chamber; the gas valve was opened to allow them to gradually lose consciousness, after which the carbon dioxide concentration was adjusted to 100%; death was confirmed by maintaining ventilation for an additional 2 min once the pinch reflex was absent and muscle tone was lost). Hypothalamic, pituitary, and ovarian tissues were harvested and stored at −80 °C for subsequent analyses.

### 2.3. Copper Content Testing

The rat hypothalamus and serum samples were dried, ashed, and digested with nitric acid (HNO_3_) until a clear solution was obtained. Copper concentrations in both hypothalamus and serum samples were analyzed in a single, unified batch using inductively coupled plasma mass spectrometry [35]. All procedures were performed in strict accordance with the manufacturer’s instructions.

### 2.4. Determination of Reproduction-Related Hormone Levels in the HPOA Axis

GnRH, FSH, LH, and E_2_ levels in rat serum were measured using ELISA kits (Mlbio, Shanghai, China), with all procedures performed strictly according to the manufacturer’s instructions. The kits demonstrated high sensitivity (limits of detection for GnRH, FSH, LH, and E_2_ were 1.0 mIU/mL, 0.1 mIU/mL, 1.0 mIU/mL, and 1.0 pmol/mL, respectively), good specificity (cross-reactivity with other related hormones < 1%), and met the study’s detection requirements-ensuring accurate measurement of low-concentration samples.

### 2.5. Total RNA Extraction and Real Time Polymerase Chain ReactionPCR

Total RNA from the hypothalamic, pituitary, and ovarian tissues was isolated using the TIANamp Tissue RNA Kit (Tiangen, Beijing, China). First-strand cDNA was synthesized from 1 μg of the total RNA using the PrimeScript RT Master Mix (Tiangen, Beijing, China) following the manufacturer’s protocol. The primer sequences were designed using the Primer 3.0 software and synthesized (Sangon, Shanghai, China). The primers used were as follows: GAPDH, F 5′-AGGTCGGTGTGAACGGATTTG-3′ and R 5′-TGTAGACCATGTAGTTGAGGTCA-3′; GnRH, F 5′-TGATGGCCGGCATTCTACTGC-3′ and R 5′-TGCCTGGCTTCCTCTTCAATC-3′; GnRHR, F 5′-GAGTGACAGTTACTTTCTTCC-3′ and R 5′-AGGAAGAAGCGTAACATTACC-3′; FSH, F 5′-TGCGGGCTACTGCTACACTAGG-3′ and R 5′-GCCAGGCAATCTTACGGTCTCG-3′; FSH-R, F 5′-GGTCTCCTTGCTGGCATTCTTGG-3′ and R 5′-CGGAATCTCTGTCACCTTGCTGTC-3′; LH-R, F 5′-TAACGAGACGCTTTATTCCGCCATC-3′ and R 5′-AGCATCTGGTTCTGGAGCACATTG-3′; LH, F 5′-GAGAATGAGTTCTGCCCAGTCTGC-3′ and R 5′-CAGTACTCGAACCATGCTAGGACAG-3′; E_2_, F 5′-CAGTCCATCCTACCCCTGGA-3′ and R 5′-TGCCGGGAACACTGTAGTTC-3′; E_2_-R, F 5′-TCCTCCTCATCCTTTCCCATATCCG-3′ and R 5′-GCATCTCCAGCAGCAGGTCATAG-3′. All primers showed acceptable efficiencies (92–108%), and GAPDH exhibited high expression stability (M = 0.28), confirming its suitability as the internal reference for reliable qRT-PCR normalization. For quantitative real-time reverse transcription polymerase chain reaction (qRT-PCR) analysis, cDNA served as the template and SYBR Green I staining (Tiangen, Beijing, China) was performed using the StepOne Plus Real-Time PCR System (Thermo Scientific, Waltham, MA, USA). The relative mRNA expression levels of each gene were calculated using the 2^−∆∆Ct^ method.

### 2.6. Transcriptomic Analysis

To elucidate the molecular mechanisms underlying copper-induced estrus onset in rats and to identify transcriptional differences, we performed RNA sequencing (RNA-seq) on hypothalamic tissues from the groups treated with 45 mg/kg of copper and the control groups. Total RNA was extracted using the TRIzol reagent (Sangon, Shanghai, China). After determining the RNA concentration, quality, and integrity using a NanoDrop spectrophotometer (Thermo Scientific, Waltham, MA, USA), the RNA Integrity Number (RIN) values of samples were as follows: C1–C6 were (9.4, 9, 9, 9.7, 9.3, 9.3, 9.7) and D1–D6 were (10, 9.4, 10, 9.7, 10, 9.5). With 6 biological replicates per group and a sequencing depth of 6G per sample, 3 μg of RNA was used as input material to prepare the sequencing library, which was purified using poly T oligonucleotide magnetic beads. The mRNA was fragmented at high temperatures using divalent cations in an Illumina-specific buffer. The first strand of cDNA was synthesized using random primers and SuperScript II, followed by synthesis of the second strand of cDNA using DNA polymerase I and RNase H. The 3′ end was adenylated using a nucleic acid exonuclease/polymerase to form flat ends and inactivate enzymes, followed by 3′ end adenylation and ligation of Illumina PE adapters; cDNA fragments of 400~500 bp were selected using the AMPure XP system (Beckman, Coulter, Brea, CA, USA), followed by 15 cycles of PCR amplification, AMPure XP purification, quantification using an Agilent 2100 bioanalyzer (Agilent Technologies, Santa, Clara, CA, USA), and sequencing on the NovaSeq 6000 platform (Illumina) (Personal Biotechnology, Shanghai, China). The transcriptome analysis included the following: ① Quality control: fastp (0.22.0) filtered raw FASTQ data to obtain clean data; ② Read alignment: HISAT2 (v2.1.0) aligned to the reference genome; ③ Expression analysis: HTSeq (v0.9.1) counted reads and normalized expression levels using FPKM; ④ Differential expression analysis: DESeq (v1.38.3) was used to screen for differentially expressed genes (DEGs) with |log^2^FoldChange| > 1 and *p*-value < 0.05. Pheatmap (v1.0.12) was used for bidirectional clustering based on Euclidean distance and complete linkage method, and a heatmap was generated; ⑤ Enrichment analysis: ClusterProfiler (v4.6.0) performed Kyoto Encyclopedia of Genes and Genomes (KEGG) enrichment (*p*-value < 0.05) and Gene Ontology (GO) enrichment (including biological process, cellular component, and molecular function categories, with *p*-value < 0.05), and GSEA (v4.1.0) plotted pathway enrichment plots; ⑥ Novel transcript analysis: StringTie (v2.2.1) assembled and aligned reads and screened for unannotated transcripts. Based on the transcriptomic analysis, qRT-PCR validation was further performed on the key DEGs in the GnRH signaling pathway and GABAergic signaling pathway in rat hypothalamic tissues. The primers used were as follows: KISS-1, F 5′-CACCCGCAGTCGCCTGATCC-3′ and R 5′-ACCTGCCTCCTGCCGTAGCG-3′; GPR54, F 5′-CTTTCCTTCTGTGCTGCGTACCC-3′ and R 5′-CGAGACCTGCTGGATGTAGTTGAC-3′; PKC, F 5′-CGAGGTGAAGAGCCACAAGT-3′ and R 5′-GGTGAACCACAAAGCTGCAG-3′; and GABA-R, F 5′-TTGGAGTGACGACCGTTCTG-3′ and R 5′-AGTCCATGGCCGTTGCATAA-3′. Meanwhile, ELISA kits were used to detect the protein contents of kisspeptin, GABA, and GnRH in the hypothalamus, so as to verify the transcriptomic results and evaluate the expression changes in related neuroendocrine factors.

### 2.7. Western Blot Analysis

Total protein was extracted from the rat hypothalamic, and hypothalamic mixed neuronal cells using a Protein Extraction Kit (Beyotime, Shanghai, China). Protein quantification was performed using a BCA Protein Assay Kit (Beyotime), followed by separation via SDS-PAGE (Yamei, Shanghai, China). Proteins were transferred to PVDF membranes (Millipore, Bedford, MA, USA), which were blocked with PBST (Servicebio, Shanghai, China) containing 5% bovine serum albumin (BSA) for 2 h. Membranes were then incubated for 2 h with primary antibodies, including GAPDH (ABClonal, Wuhan, China, A19056), PKC (ABClonal, A24003), p-PKC (ABClonal, AP0191), KISS-1 (Wanleibio, Shenyang, China, WL02651), GPR54 (ABClonal, A2967), and GABA-R (KALANG, Shanghai, China), with dilution ratios following the manufacturers’ instructions. After incubation with horseradish peroxidase-conjugated goat anti-rabbit IgG (H + L) (ABClonal, AS014, 1:5000 dilution) for 1 h, protein blots were developed and imaged using a QuickChemi 5200 chemiluminescence imaging system (Monad, Shanghai, China, GD50202).

### 2.8. Isolation, Culture, and Identification of Hypothalamic Mixed Neurons

The experiment utilized 10 pregnant female SD rats (gestational day 17) purchased from Liaoning Changsheng Biotechnology Co., Ltd. (Shenyang, China), and these rats were of the same strain as those used in the in vivo experiment. Following euthanasia, fetuses were extracted on a sterile workbench and decapitated; the brain tissues were rapidly harvested and immersed in ice-cold HESS (Servicebio, Shanghai, China). The meninges and blood clots were carefully removed, and the hypothalamus was isolated (anterior–posterior boundaries: optic chiasm to the mammillary body; lateral boundaries: hypothalamic sulci; depth, approximately 1.5 mm). Brain tissues were rinsed with D-Hanks solution (Servicebio, Shanghai, China), minced into 1 × 1 × 1 mm fragments, and incubated in 0.125% trypsin (Thermo Scientific, Waltham, MA, USA) at 37 °C for 10 min. The digested tissue was resuspended in DMEM/F12 medium (1:1 ratio, containing 10% fetal bovine serum; Thermo Scientific, Waltham, MA, USA), filtered through a 100 µ-mesh cell strainer, and centrifuged at 1000 rpm for 5 min to collect the cell pellet. Cells were seeded at a density of 5 × 105 cells/cm^2^ onto glass slides pre-coated with poly-L-lysine (WHB, Shanghai, China) and cultured in a 37 °C incubator with 5% CO_2_. After 24 h, the medium was replaced with neurobasal medium (Thermo Scientific, Waltham, MA, USA) supplemented with 2% B27 and 1% GlutaMAX, and the medium was changed every 2~3 d. Cell morphology was observed under an inverted microscope on days 1, 3, 5, 7, and 9. After 7 d of culture, NSE immunofluorescence staining was performed. The cells were placed in culture plates, washed three times with phosphate-buffered saline (PBS), fixed with 4% paraformaldehyde for 15 min, and washed three times with PBS. The cells were permeabilized with 0.5% Triton X-100 (Beyotime, Shanghai, China) at room temperature (22–24 °C) for 20 min, rinsed thrice with PBS, and blocked with normal goat serum (Boster, Wuhan, China) at room temperature for 30 min. After removing the blocking solution, the primary antibody NSE (Affinity, Cincinnati, OH, USA, AF5473) was added and incubated overnight at 4 °C in a humidified chamber. The following day, the plates were washed three times with PBS and Goat anti-rabbit Cy3 (Proteintech, Rosemont, IL, USA, SA00009-2) was added, followed by incubation at 20~37 °C for 1 h and subsequent three washes with PBS. DAPI (Beyotime, Shanghai, China) was added, and the cells were incubated in the dark for 5 min to stain the nuclei, followed by washing four times with PBS. Finally, the cells were mounted with mounting medium containing an anti-fluorescence quencher and images were acquired under a fluorescence microscope.

### 2.9. Screening for the Optimal Copper Concentration and Concentrations of PKC Activators and Inhibitors for Hypothalamic Mixed Neurons

Based on the in vivo copper ion content in the hypothalamus of rats, the molar concentrations of copper ions in the in vitro cell culture medium were calculated and designed in gradients, and primary cultured hypothalamic mixed neurons were subsequently divided into six groups, with copper ion concentrations of 0 (control), 15.6, 31.2, 46.8, 62.4, and 78 μmol/L added to the culture medium in the form of CuSO_4_·5H_2_O (Sigma, St. Luois, MO, USA). Cell viability was assessed using the CCK-8 assay kit (Sangon, Shanghai, China). Ten microliters of the reagent were added to each well of a 96-well plate and incubated for 2 h. The GnRH levels in the culture medium were measured using a rat GnRH ELISA kit (MIbio, Shanghai, China). The assay was performed according to the manufacturer’s instructions, and the absorbance was read at 450 nm. GnRH concentration was calculated using a standard curve. After identifying the optimum copper concentration, further screening was performed to determine the optimal concentrations of the PKC activator (PMA) and the inhibitor (chelerythrine). All reagents were purchased from MedChemExpress (Monmouth Junction, NJ, USA). Using hypothalamic mixed neurons treated with the optimal copper concentration, different concentration gradients of PMA (0, 10, 20, 40, and 80 nM) and chelerythrine (0, 1, 2, 4, and 8 μM) were set for intervention, following the methodology reported by Takahito et al. [36]. Cell viability was assessed using the CCK-8 assay kit (Sangon, Shanghai, China). Western blot analysis was used to determine the levels of PKC in each treatment group, which reflected changes in PKC activity. This enabled the screening of the optimal concentrations of PMA and chelerythrine which significantly regulate PKC activity without inducing significant cellular toxicity, thus paving the way for subsequent mechanistic validation experiments.

### 2.10. Statistical Analyses

Statistical analyses were performed using IBM SPSS Statistics 26. Following tests for normality (Shapiro–Wilk test) and homogeneity of variance (Levene’s test), intergroup differences were analyzed using one-way analysis of variance, followed by Duncan’s multiple range test. Data are presented as mean ± standard deviation, and statistical significance was set at *p* < 0.05.

## 3. Results

### 3.1. Identification of Rat Estrus and Effects of Copper on Puberty Age

**Figure 1:**
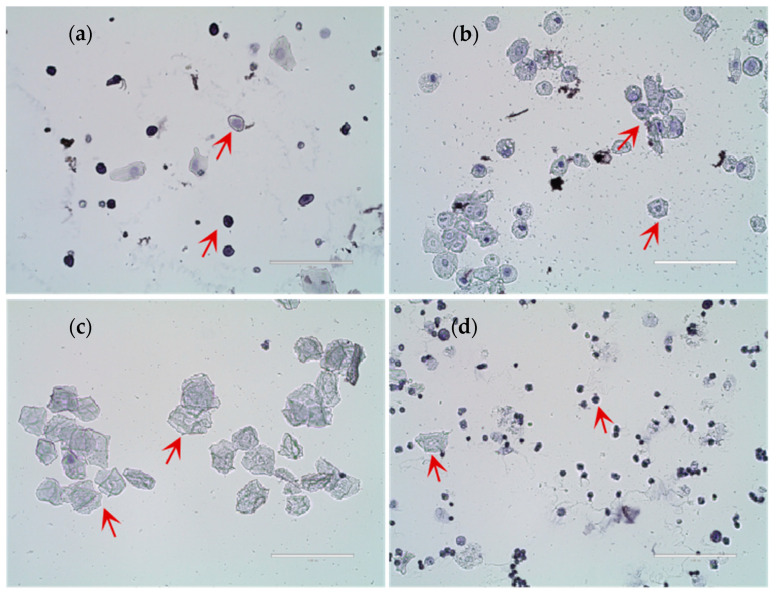
Results of rat estrous cycle identification. (**a**) Diestrus; (**b**) Proestrus; (**c**) Estrus; (**d**) Metestrus (*n* = 10). Scale bar = 100 μm.

**Figure 2:**
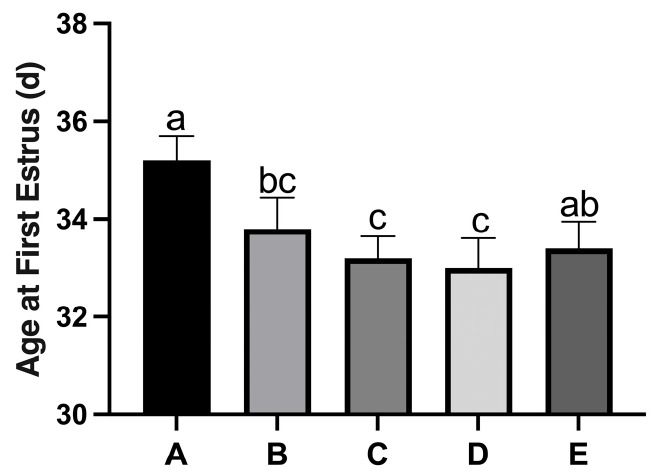
Effect of copper on the age at first estrus in rats. Group A, control diet group; Group B, control diet + 15 mg/kg Cu; Group C, control diet + 30 mg/kg Cu; Group D, control diet + 45 mg/kg Cu; Group E, control diet + 60 mg/kg Cu. (*n* = 10) Note: Within the same parameter, different letters (a, b, and c) indicate significant differences (*p* < 0.05), while the same letters or no letters indicate no significant differences (*p* > 0.05). This notation applies to subsequent figures.

Figure 1 shows the cellular characteristics of different estrous cycle stages in rats based on hematoxylin-stained vaginal smears. (a) Diestrus was characterized by predominantly leukocytes with scattered epithelial cells; (b) Proestrus exhibited mainly nucleated epithelial cells with visible nuclei; (c) Estrus displayed numerous anucleated, cornified squamous epithelial cells; (d) Metestrus showed a transition pattern with leukocytes and residual cornified cells. To ensure experimental consistency, all sample collections were performed during the second proestrus phase. With increasing dietary copper supplementation, the age at first estrus was significantly lower than that of the control Group A (Figure 2). The age at first estrus was the earliest in Group D, which was significantly different from those of Groups A and E, but not significantly different from those of Groups B and C.

### 3.2. Detection of Copper Content in the Hypothalamus and Serum of Rats

Copper levels in the rat hypothalamus and serum tended to increase non-significantly with increasing dietary copper supplementation (Table 1).

### 3.3. Effects of Copper on the Secretion of Reproduction-Related Hormones in the HPOA Axis During Puberty Initiation in Rats

The levels of reproductive hormones (GnRH, FSH, LH, and E_2_) in rat serum tended to initially increase and then decrease with increasing dietary copper supplementation (Table 2). Among these, the GnRH level in Group D was the highest and was significantly higher than those in Groups A, B, and E, but the difference with Group C was not significant. FSH levels in Group C were significantly higher than those in Groups A, B, D, and E, whereas no significant differences were observed among Groups A, B, D, and E. The LH levels in Groups C and D were significantly higher than those in Groups A, B, and E, with no significant differences between Groups C and D. Additionally, the LH level in Group B was significantly higher than those in Groups A and E, and the LH level in Group A was significantly higher than that in Group E. The E_2_ levels in Groups C and D were significantly higher than those in Groups A, B, and E, with no significant differences between Groups C and D or among Groups A, B, and E.

### 3.4. Effects of Copper on the Relative mRNA Expression Levels of Reproduction-Related Hormones in the HPOA Axis During Puberty Initiation in Rats

**Figure 3:**
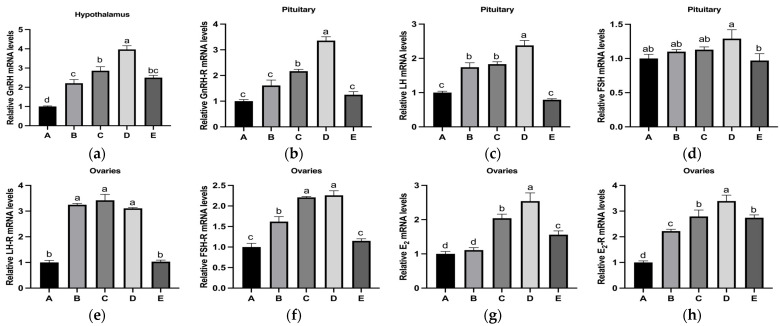
Effects of copper supplementation on the mRNA levels of reproduction-related hormones in the HPOA axis during puberty initiation in rats. (**a**) Relative GnRH mRNA levels. (**b**) Relative GnRH-R mRNA levels. (**c**) Relative LH mRNA levels. (**d**) Relative FSH mRNA levels. (**e**) Relative LH-R mRNA levels. (**f**) Relative FSH-R mRNA levels. (**g**) Relative E_2_ mRNA levels. (**h**) Relative E_2_-R mRNA levels (*n* = 6) Abbreviations: GnRH-R, gonadotropin-releasing hormone receptor; LH-R, luteinizing hormone receptor; FSH-R, follicle-stimulating hormone receptor; E_2_-R, estradiol receptor. Note: Within the same parameter, different letters (a, b, c, and d) indicate significant differences (*p* < 0.05), whereas the same letters or no letters indicate no significant difference (*p* > 0.05).

The mRNA expression levels of reproductive hormones and their receptors (GnRH, GnRH-R, LH, FSH, LH-R, FSH-R, E_2_, and E_2_-R) in the HPOA axis tended to initially increase and then decrease with increasing dietary copper supplementation (Figure 3). Specifically, the mRNA levels of GnRH in the hypothalamus and GnRH-R and LH in the pituitary gland reached their peaks in Group D, which were significantly higher than those in the other groups (Figure 3a–c). The FSH mRNA levels in the pituitary glands of Group D were significantly higher than those in Group E, but there was no significant difference compared to those in Groups A, B, and C (Figure 3d). The ovarian LHR mRNA levels in Groups B, C, and D were significantly higher than those in Groups A and E, whereas no significant differences were observed among Groups B, C, and D (Figure 3e). Groups C and D showed significantly higher ovarian FSH-R mRNA levels than those in Groups A, B, and E, with no significant difference between Groups C and D (Figure 3f). Additionally, the ovarian mRNA levels of E_2_ and E_2_-R showed a similar trend for changes in hypothalamic GnRH mRNA levels (Figure 3g–h).

### 3.5. Transcriptome Analysis and Validation

**Figure 4:**
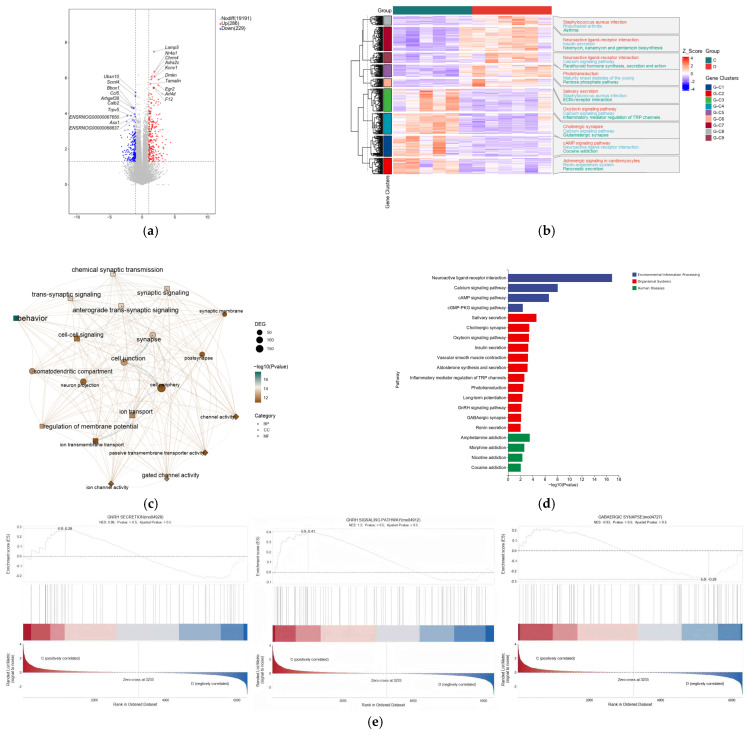
Transcriptomic analysis of the mechanism by which dietary copper affects the onset of puberty. (**a**) volcano map of mRNA expression profiles. (**b**) The top 50 of differentially expressed genes (DGEs). (**c**) The top 10 enriched Kyoto Encyclopedia of Genes and Genomes (KEGG) pathway of DEGs. (**d**) The top 10 enriched Gene Ontology (GO) pathway of the DEGs sorted by significance in biological process (BP), cellular component (CC) and molecular function (MF). (**e**) The results were enriched to GnRH signaling pathway, GABAergic synapse and GnRH secretion (*n* = 6).

**Figure 5:**
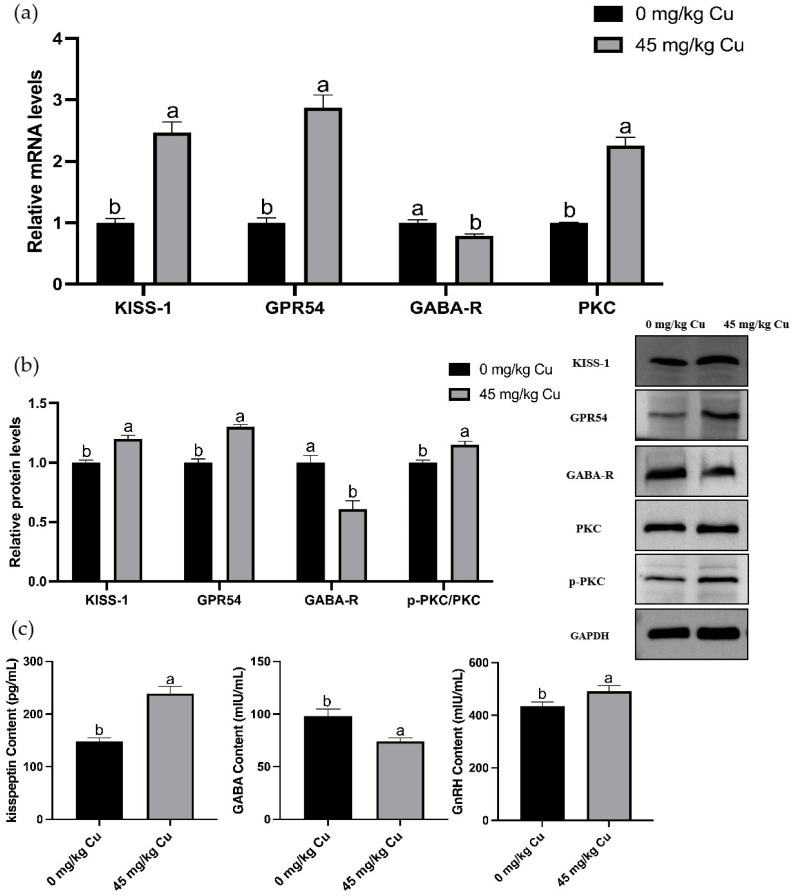
Validation of differentially expressed genes in rat hypothalamus based on omics findings. (**a**) Relative KISS-1, GPR54, PKC, GABA-R mRNA levels. (**b**) Relative p-PKC/PKC, GABA-R, KISS-1, GPR54 protein levels. (**c**) Hypothalamus kisspeptin, GABA, GnRH content. Abbreviations: KISS-1, kisspeptin-1; GPR54, protein-coupled receptor 54; PKC, Protein Kinase C; GABA, γ-aminobutyric acid; GABA-R, γ-aminobutyric acid receptor (*n* = 6) Note: Within the same parameter, different letters (a and b) indicate significant differences (*p* < 0.05), whereas the same letters or no letters indicate no significant difference (*p* > 0.05).

The results revealed that copper treatment significantly altered the hypothalamic gene expression profile, with 515 differentially expressed genes identified, of which 286 were upregulated and 229 were downregulated (Figure 4). GO and KEGG pathway enrichment analyses of these DEGs revealed that they were significantly enriched in multiple KEGG signaling pathways, including neuroactive ligand-receptor interaction, calcium signaling, GnRH signaling, GABAergic synapses, cAMP signaling, and cGMP-PKG signaling (Figure 4d). The top 20 enriched GO terms included changes in biological processes (BP), cellular components (CC), and molecular functions (MF) such as behavioral processes, synaptic transmission, brain development, neuropeptide signaling pathways, central nervous system development, feeding behavior, and neuropeptide hormone activity (Figure 4c). Further analysis revealed that, compared with the control group (Figure 4e), the upregulated gene KISS-1 was enriched in the GnRH pathway, while the downregulated gene GABA_a_ and the upregulated gene PKC were co-enriched in the GABA signaling pathway. Validation using qRT-PCR showed that copper treatment upregulated the mRNA and protein levels of GnRH-related markers, which was specifically manifested as follows: the contents of kisspeptin and GnRH in rat hypothalamic tissues significantly increased (Figure 5c); the mRNA and protein expressions of KISS-1, GPR54, and PKC significantly increased, with a significant elevation in PKC phosphorylation level; concurrently, the GABA content in the hypothalamus and the relative mRNA and protein expression levels of GABA-R were significantly reduced (Figure 5a,b).

### 3.6. Identification of Rat Hypothalamic Neuronal Cells

**Figure 6:**
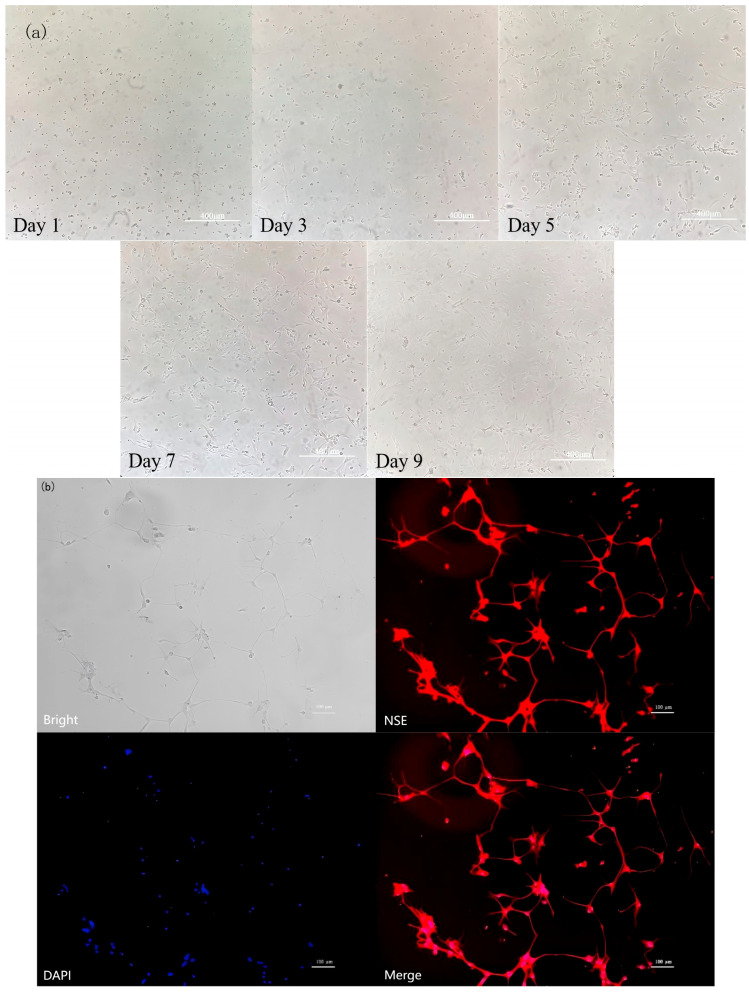
Identification of rat hypothalamic mixed neuronal cells. (**a**) The dynamic growth of primary cultured rat hypothalamic mixed neurons from day 1 to day 9. Scale bar = 400 μm. (**b**) NSE immunofluorescence staining (*n* = 3). Scale bar = 100 μm.

**Figure 7:**
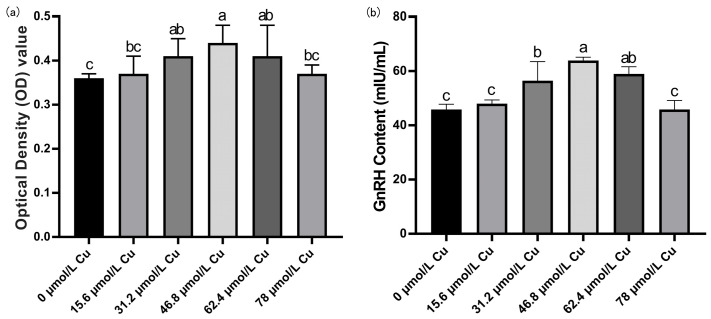
Screening for the optimal copper concentration in hypothalamic mixed neurons. (**a**) Cell viability OD value detection results. (**b**) GnRH content in cell culture medium (*n* = 3). Note: Within the same parameter, different letters (a, b, and c) indicate significant differences (*p* < 0.05), whereas the same letters or no letters indicate no significant difference (*p* > 0.05).

Figure 6 shows the dynamic growth of primary cultured rat hypothalamic mixed neurons from day 1 to day 9. On day 1, the cells were suspended and began to adhere to the wall; on day 3, the adherent cells appeared as short spindle-shaped cells with extended protrusions; on day 5, a network structure was formed; and from day 7 to day 9, axons and dendrites were highly interwoven. Figure 7b also shows that after 7 d of culture; the cells were subjected to NSE immunofluorescence staining. The red fluorescence labeled with Cy3 and the blue cell nuclei counterstained with DAPI showed clear localization with no nonspecific staining, confirming that the cells were predominantly neurons. These results demonstrated that a stable culture system can be used to obtain high-purity hypothalamic neurons.

### 3.7. Screening of Optimal Copper Concentration

The viability of rat hypothalamic mixed neurons and GnRH levels in the culture medium initially increased and then decreased with increasing copper concentration (Figure 7). Compared with the control group, at a copper concentration of 46.8 μmol/L, cell viability and GnRH levels in the culture medium were the highest. Therefore, 46.8 μmol/L copper was selected as the optimal concentration for subsequent experiments.

### 3.8. Effects of PMA and Chelerythrine Treatments on Copper-Regulated GABA and GnRH Pathways in Hypothalamic Mixed Neurons

**Figure 8:**
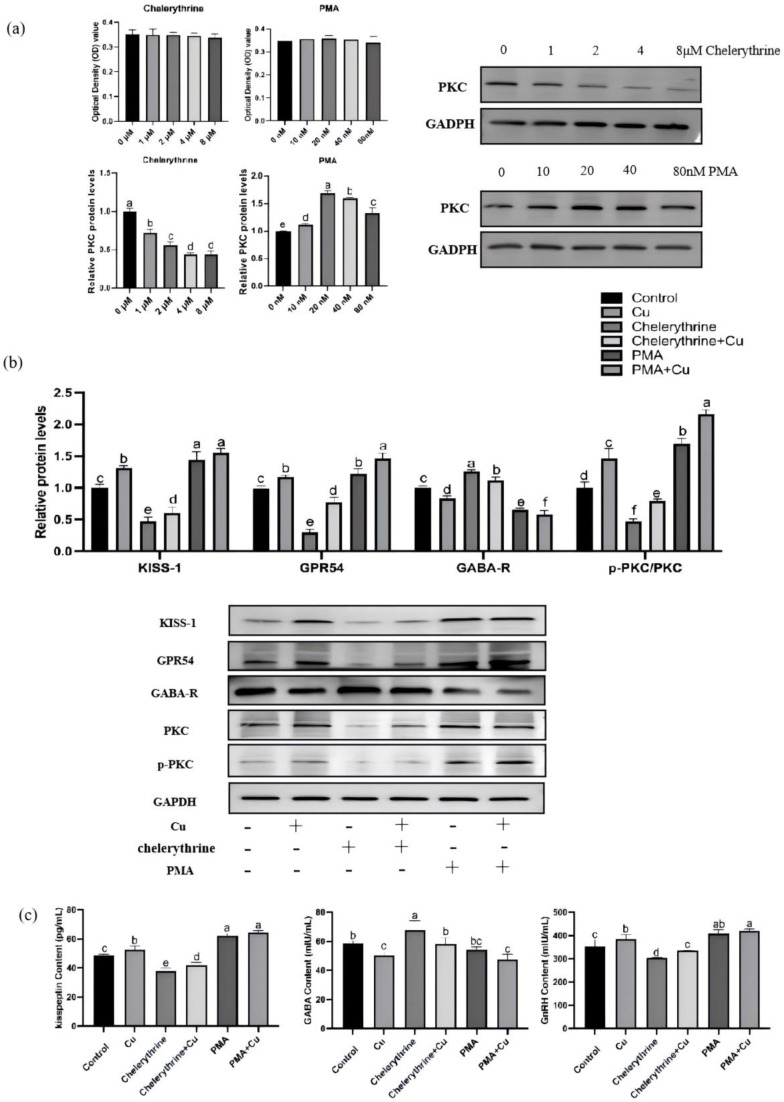
Effects of PMA and chelerythrine treatments on copper-regulated GABA and GnRH pathways in hypothalamic mixed neurons. (**a**) Concentration screening of PKC activators and inhibitors. (**b**) Relative KISS-1, GPR54, GABA-R, p-PKC/PKC protein levels. (**c**) Hypothalamus kisspeptin, GABA, GnRH content (*n* = 3). Note: Within the same parameter, different letters (a, b, c, d, e and f) indicate significant differences (*p* < 0.05), whereas the same letters or no letters indicate no significant difference (*p* > 0.05).

The results in Figure 8a showed that treatment with different doses of PMA or chelerythrine did not significantly affect the viability of primary cultured rat hypothalamic mixed neurons. The results showed that compared with the control group, the PKC protein expression in the chelerythrine-treated groups gradually decreased with increasing dose, and thus the intermediate concentration of 2 μM was selected for subsequent experiments. In the PMA-treated groups, the PKC protein expression first increased and then decreased; among them, the PKC protein level in the 20 nM PMA-treated group was significantly higher than that in other groups, so this concentration was determined as the one to be used in subsequent experiments.

As shown in Figure 8b,c, in rat hypothalamic mixed neurons treated with 46.8 μmol/L copper, the protein expression of KISS-1 and GPR54, PKC phosphorylation level, and the contents of kisspeptin and GnRH were all significantly increased, while GABA-R protein and GABA were significantly decreased. This trend of change was consistent with the results observed in in vivo hypothalamic tissues. The combined application of copper and chelerythrine could partially counteract the inhibitory effects induced by chelerythrine treatment alone—specifically, the decreases in PKC phosphorylation, KISS-1/GPR54 expression, and kisspeptin/GnRH contents were partially restored, while the abnormal increases in GABA-R expression and GABA level were significantly improved. In contrast to the action direction of chelerythrine, PMA could significantly activate the PKC signal, exhibiting effects including increased PKC phosphorylation, enhanced KISS-1/GPR54 expression and kisspeptin/GnRH contents, as well as inhibited GABA-R expression and reduced GABA level; when co-treated with copper, these pro-activation effects were further enhanced, showing a tendency of synergistic effect.

## 4. Discussion

This study was designed to investigate the role of dietary copper in regulating puberty onset in female SD rats and to elucidate the underlying neuroendocrine mechanisms. The central finding is that a dietary copper level of 45 mg/kg significantly advances puberty initiation without inducing pathological precocity, indicating that copper can serve as an important nutritional regulator of reproductive development during puberty. This effect is mediated through coordinated modulation of the HPOA axis, particularly via activation of the KISS-1/GPR54-GnRH system and suppression of GABAergic inhibition, with PKC emerging as a key signaling node.

### 4.1. Dose-Dependent Regulation of Puberty by Copper

Previous studies have shown that female rats typically enter puberty between postnatal days 32 and 38, although the exact timing may vary depending on factors such as strain differences and housing conditions [37,38]. In the present study, compared with the control group, dietary supplementation with 45 mg/kg copper significantly advanced the onset of puberty in rats, indicating that appropriate copper supplementation can effectively promote the initiation of puberty. However, the magnitude of this advancement remained within the normal physiological range, suggesting that such a change represents a moderate precocious puberty that does not exceed the normal developmental window and will not induce pathological precocious puberty. Prior research has demonstrated that the serum copper concentration in rats usually ranges from 1 to 2 mg/L under normal physiological conditions, which is comparable to the serum copper level in humans [39,40,41]. In this experiment, the serum copper concentration of rats in the control group was approximately 1.2 mg/L; with the increase in dietary copper supplementation, the serum copper level increased in a dose-dependent manner but did not exceed the normal physiological range, which not only indicates that the copper supplementation dose used in this study is safe and within the physiologically regulatable range (and will not cause copper overload or copper toxicity) but also reflects the body’s precise regulation of copper homeostasis. Furthermore, the correlation between the moderate elevation of serum copper levels and the advanced initiation of puberty suggests that copper may regulate the function of the HPOA axis by maintaining dynamic changes within the normal physiological concentration range, thereby influencing the process of pubertal development.

### 4.2. Activation of the HPOA Axis by Copper

Further mechanistic studies revealed the precise regulatory effect of copper on the HPOA axis. Previous research has shown that intracerebroventricular injection of copper sulfate into rats and rabbits significantly increases ovulation rates [42]. The core mechanism lies in the fact that copper ions released by hypothalamic neurons can precisely regulate the secretion of LH and FSH—two gonadotropins that are crucial for initiating and maintaining reproductive function [43]. Of particular importance, a study by Schvartz et al. [44] confirmed that copper ions exert time- and dose-dependent stimulatory effects on LH release from cultured rat pituitary cells, and this effect is primarily mediated by the GnRH pathway. As a key regulator of the HPOA axis, GnRH is synthesized by specific hypothalamic neurons and secreted in a pulsatile manner [45,46,47]. Optimal copper content maintains GnRH neuron function and HPOA axis homeostasis, whereas copper deficiency or excess disrupts GnRH secretion and receptor activity, leading to imbalanced LH and FSH secretion, impaired estrogen synthesis, and disrupted reproductive cycles [48,49]. These findings align closely with our observations in our study that serum and HPOA axis levels of GnRH, LH, FSH, E_2_, and their respective receptors initially increase and then decrease with increasing dietary copper levels, mirroring the changes in age at puberty onset. Meanwhile, in the serum of rats, we observed that, unlike the other three hormones, FSH reached its secretion peak at a dietary copper addition of 30 mg/kg. This may be because this dose most effectively activates FSH release pathways while not excessively inducing the activity of its specific metabolic enzymes, thereby preventing the rapid degradation of serum FSH. Additionally, the unique sensitivity of FSH to GnRH pulse frequency and feedback regulation thresholds may also be involved, and the specific mechanism requires further research and verification. Thus, appropriate dietary copper supplementation positively regulates puberty initiation by stimulating hypothalamic GnRH secretion and receptor expression, promoting pituitary LH/FSH release, and enhancing ovarian E_2_ synthesis. Furthermore, these results underscore the bidirectional dose-dependent effects of copper on reproductive endocrinology.

### 4.3. Transcriptomic Insights: Involvement of GnRH and GABA Signaling Pathways

In this study, we employed RNA sequencing to compare hypothalamic transcriptomic differences in rats under varying dietary copper levels, performed functional enrichment analyses, and validated key signaling pathways to explore the potential mechanisms by which copper promotes puberty initiation. Functional GO annotation and KEGG pathway analysis of all DEGs revealed that both the GnRH signaling pathway and GABA signaling pathway were significantly enriched in the copper-supplemented groups among metabolism- and organismal system-related pathways, providing critical molecular evidence for copper-mediated regulation of GnRH release. Further results showed that KISS-1, a core regulatory gene of the GnRH signaling pathway, was significantly upregulated in the copper-treated groups, suggesting that copper may enhance GnRH neuronal activity by activating the KISS-1/GPR54 system, thereby facilitating the onset of puberty. This system acts as a molecular switch which governs the initiation and homeostasis of the mammalian reproductive axis. Hypothalamic KISS-1 is primarily expressed in neuronal populations within the arcuate nucleus (ARC) and anteroventral periventricular nucleus (AVPV), and its transcription product, kisspeptin, directly activates GnRH neurons to regulate reproductive function. Anatomical studies have confirmed direct synaptic connections between kisspeptin-immunoreactive fibers and GnRH neurons [50,51], while perfusing GnRH neurons with kisspeptin effectively activates these neurons and increases their firing frequency [52,53,54,55]. Additionally, this neuroendocrine regulatory mechanism not only governs gonadotropin release during puberty but also maintains reproductive cycle rhythms in adulthood [56,57]. In this study, dietary supplementation with 45 mg/kg Cu significantly upregulated the hypothalamic mRNA and protein expression of KISS-1 and GPR54 in rats, concurrent with increased kisspeptin levels in the hypothalamic tissues. These changes were consistent with fluctuations in the key genes of the HPOA axis, further confirming the positive regulatory effect of copper on the KISS-1/GPR54 system. Furthermore, studies in multiple mammalian species, including mice [58], pigs [59], sheep [60], primates [52], have indicated that changes in energy metabolic status (e.g., glucose/fatty acid metabolism or fluctuations in ATP/AMP ratios) can drive GnRH neuron activity by activating the hypothalamic KISS-1/GPR54 system, regulating the HPOA axis, and triggering puberty initiation. Our transcriptomic analysis also revealed that copper was significantly enriched in multiple energy metabolism-related pathways, including adipocyte lipolysis, gluconeogenesis, and glycolysis. As a regulator of energy metabolism, copper deficiency induces excessive lipolysis and reduces body fat mass in rats [61], and copper participates in hepatic gluconeogenesis and skeletal muscle fatty acid oxidation [62]. Meanwhile, copper-induced energy metabolism imbalance can trigger metabolic syndrome and reproductive disorders (e.g., delayed puberty) [63,64], although the specific molecular mechanisms remain unclear and warrant further investigation.

In the differential gene analysis of the GABA signaling pathway in this study, it was found that copper supplementation significantly downregulated the expression level of the GABA_a_ receptor. GABA, a key inhibitory neurotransmitter in the central nervous system, is synthesized from glutamate by glutamic acid decarboxylase (GAD). It binds to GABA_a_/_b_ receptors on the postsynaptic membrane, inducing postsynaptic membrane hyperpolarization and thereby inhibiting excessive neuronal excitability. GABA is involved in regulating nervous system functions and processes, such as reproduction, energy metabolism, and intestinal immunity [65,66,67,68]. The effects of GABA on GnRH neurons exhibit developmental stage specificity, and GABA inhibits GnRH neurons to maintain the reproductive axis in a quiescent state. During puberty onset, this inhibitory effect weakens, leading to the activation of GnRH neurons, which in turn affects the initiation of puberty [69,70]. Our data revealed that with optimal copper supplementation, GABA levels in the serum and hypothalamus as well as hypothalamic GABA receptor expression were significantly reduced during puberty initiation, confirming that copper inhibited the GABA system at this stage. KISS-1 and GABA neurons are significantly co-expressed in the rostral periventricular region of the third ventricle (RP3V), which regulates GnRH neurons in a frequency-dependent manner. Low-frequency GABA release induces transient excitation, while high-frequency kisspeptin release triggers sustained activation, which precisely regulates GnRH pulsatile secretion during ovulation [71,72,73]. From a neuroendocrine network perspective, copper-mediated reduction in GABA levels may promote GnRH release through the following two mechanisms: (1) relieving the tonic inhibition of GABA on RP3V KISS-1/GABA neurons, thus enhancing kisspeptin-mediated activation under high-frequency stimulation; and (2) direct reduction in GABA-mediated inhibition of GnRH neurons, thus increasing the sensitivity of the KISS-1/GPR54 signaling pathway, thereby driving puberty onset. Additionally, GABA metabolites participate in the tricarboxylic acid cycle via GABA transaminase (GABA-T) and influence energy metabolism by regulating appetite (elevated GABA levels inhibit feeding, whereas reduced levels promote feeding) [74,75,76]. Considering that copper reduces serum leptin and somatostatin levels while increasing ghrelin levels [32,77], these results suggest that copper may coordinate reproductive axis activation and energy metabolism by modulating the GABA signaling pathway, thus highlighting its potential role as a metabolic-reproductive regulatory hub.

### 4.4. PKC as a Critical Convergence Point in Copper-Mediated Puberty Regulation

In the GABA signaling pathway, copper treatment significantly upregulates the expression of PKC. PKC can mediate the release of gonadotropins induced by GnRH in a variety of animals, and this pathway is enriched in both the GnRH and KISS-1/GPR54 systems, suggesting that PKC may be a critical node linking copper-regulated GABA and KISS-1/GPR54-GnRH signaling pathways. In the context of reproductive regulation, PKCδ deficiency weakens the fertilizing ability of male mouse sperm to penetrate the zona pellucida and reduces female fertility [78], while PKC activation promotes the resumption of bovine oocyte meiosis and increases the ratio of double-pronuclear oocytes [79]. A copper-deficient diet downregulates the expression of multiple PKC isoforms and increases the risk of colorectal cancer [29,80]. In female mice on a low-copper diet, cytosolic levels of PKCβ in the hypothalamus and PKCγ in the cerebellum of offspring were significantly lower than those in the control group [32]. Consistently, our study showed that in the copper-supplemented groups, hypothalamic PKC mRNA expression and protein phosphorylation levels significantly increased during puberty, thus confirming that copper modulates hypothalamic PKC expression. Additionally, PKC is involved in mediating GnRH-induced gonadotropin release in both mammals and non-mammals [81,82]. PKC is activated upon neuronal stimulation to regulate neurotransmitter release, promoting the fusion of synaptic vesicles with the presynaptic membrane through the phosphorylation of presynaptic membrane-associated proteins, thereby mediating neurotransmitter release and facilitating intercellular communication [83,84]. PKC activation downregulates GABAA receptor-mediated tonic inhibition in the hippocampal dentate gyrus granule cells and dorsolateral thalamic relay neurons, whereas PKC inhibition enhances this inhibitory effect. Conversely, GABA receptor activation can indirectly inhibit PKC activity by suppressing the cAMP-PKA pathway, forming a “PKC-GABA bidirectional regulatory module.” Thus, PKC-mediated phosphorylation is a key mechanism regulating GABAA receptor tonic inhibition and maintaining the neurotransmitter balance [85]. In summary, as a member of the serine/threonine protein kinase family, alterations in PKC activity may mediate the cascading regulation of the neuroendocrine system through the phosphorylation of downstream target proteins, thereby regulating the reproductive axis.

### 4.5. In Vitro Validation of the PKC-Centered Regulatory Pathway

To validate the in vivo findings, primary cultures of rat hypothalamic mixed neurons were used for in vitro experiments in this study. Multi-parameter assessment identified 46.8 μmol/L as the optimal copper concentration for promoting GnRH secretion. At this concentration, copper treatment significantly increased the phosphorylation level of PKC, protein expression levels of KISS-1 and GPR54, and contents of kisspeptin and GnRH in rat hypothalamic mixed neurons, while decreasing the protein level of GABA-R and content of GABA. This result was consistent with the in vivo trend. Further intervention with the PKC-specific inhibitor chelerythrine led to changes in the above indicators opposite to those in the copper-treated group, and this inhibitory effect could be partially reversed by copper. In contrast, treatment with the PKC activator PMA resulted in changes opposite to those induced by chelerythrine, and co-treatment with copper and PMA could enhance the effect of PMA. These in vitro data are highly consistent with the in vivo results, strongly confirming that PKC plays a key role in the copper-mediated regulation of GABA neurotransmission, KISS-1/GPR54-GnRH signaling activation, and puberty onset. To mitigate potential off-target effects of PMA and chelerythrine, we used low doses of both compounds (consistent with previously reported non-toxic concentrations) and strictly controlled the treatment duration in all in vitro experiments. Additionally, PKC contributes to energy metabolism regulation by inhibiting acetyl-CoA carboxylase (a rate-limiting enzyme in fatty acid synthesis), modulating lipoprotein receptor function, and activating adipocyte lipolysis, thereby maintaining systemic energy homeostasis [80,86]. PKCβ is induced in white adipose tissue by a high-fat diet, and its deficiency renders transgenic mice resistant to high-fat diet-induced obesity and hepatic steatosis. Atypical PKCλ/ι subtypes act as key regulators of mitochondrial function in mouse embryonic stem cells; their deficiency leads to mitochondrial maturation defects and a metabolic shift toward glycolysis [87,88]. Thus, PKC has multisystem regulatory functions, particularly potential crosstalk between energy metabolism and the neuroendocrine system. Copper ions may directly target PKC (e.g., inducing conformational changes, exposing phosphorylation sites, or interfering with phosphatidylserine binding) or indirectly regulate pathways such as Ca^2+^/MAPK/PLC-DAG [31,89], thereby altering PKC activity and neuroendocrine signaling to influence systemic energy metabolism. Conversely, whether metabolic states regulate pubertal development and reproductive function by influencing copper homeostasis and PKC phosphorylation via feedback mechanisms represents a potential key direction for regulating reproduction.

### 4.6. Study Limitations and Future Directions

The findings of this study attempt to fill the gap in the mechanism underlying copper regulation of puberty onset, provide preclinical evidence for the subsequent exploration of copper nutritional intervention strategies in human adolescents, and offer a new nutritional strategy perspective for addressing abnormal puberty issues. However, this study still has the following limitations, and further research can be conducted around these directions: First, the study focused only on the puberty initiation stage and did not evaluate the long-term effects of copper on reproductive function in adulthood, such as the estrous cycle, ovarian reserve, and fertility. Given that abnormal puberty is often associated with adult reproductive diseases, longitudinal follow-up experiments will be conducted in subsequent studies to systematically assess the impact of copper on reproductive health across different life stages. Second, while SD rats are a commonly used model, there are interspecies differences in copper metabolism and HPOA axis regulation. In the future, attempts will be made to use human induced pluripotent stem cell (iPSC)-derived hypothalamic-like cells or neuroorganoid models to validate key pathways, thereby enhancing clinical relevance. Finally, transcriptomic data show enrichment of energy metabolism pathways, suggesting that copper may be involved in “metabolism-reproduction” integration. However, the interactive relationships between factors such as leptin, ghrelin, kisspeptin, and GABA remain unclear. In subsequent studies, we will combine metabolic interventions (e.g., energy restriction or high-fat diet) and block specific signals via central administration (e.g., GABA-R antagonists, KISS-1 siRNA) to clarify the regulatory role of PKC in this process.

## 5. Conclusions

In conclusion, this study is the first to confirm that a copper intake of 45 mg/kg in SD rats can promote puberty. In this animal model, the underlying mechanism involves activation of the hypothalamic PKC pathway to mediate copper-induced cascade regulation of the neuroendocrine system. This process inhibits GABAergic neurotransmission and GABA receptor expression to alleviate the inhibitory state of the reproductive axis. Simultaneously, it activates the KISS-1/GPR54-GnRH system, promotes pulsatile LH and FSH secretion, effectively regulates HPOA axis activity, and ultimately facilitates puberty in rats.

## Figures and Tables

**Table 1:** Copper content in serum and the hypothalamus.

Items	A	B	C	D	E
Hypothalamus (mg/kg)	2.77 ± 0.07	2.91 ± 0.29	2.92 ± 0.30	3.10 ± 0.31	3.15 ± 0.33
Serum (mg/L)	1.20 ± 0.11	1.43 ± 0.15	1.59 ± 0.05	1.64 ± 0.23	1.74 ± 0.23

**Table 2:** Serum levels of estrus related hormones.

Items	A	B	C	D	E
GnRH (mIU/mL)	63.23 ± 2.90 ^d^	68.48 ± 3.41 ^b,c^	70.69 ± 6.06 ^a,b^	74.71 ± 3.87 ^a^	65.08 ± 2.63 ^c,d^
FSH (mIU/mL)	9.24 ± 0.13 ^b^	9.43 ± 0.22 ^b^	10.05 ± 0.41 ^a^	9.43 ± 0.21 ^b^	9.17 ± 0.28 ^b^
LH (mIU/mL)	30.73 ± 3.31 ^c^	35.41 ± 1.11 ^b^	39.24 ± 2.86 ^a^	40.05 ± 1.23 ^a^	24.8 ± 1.14 ^d^
E_2_ (pmol/mL)	45.69 ± 2.60 ^b^	45.27 ± 3.95 ^b^	49.14 ± 2.78 ^a^	49.56 ± 1.55 ^a^	45.41 ± 1.20 ^b^

Abbreviations: GnRH, gonadotropin releasing hormone; FSH, follicle-stimulating hormone; LH, luteinizing hormone; E_2_, estradiol. (*n* = 6) Note: Within the same parameter, different letters (a, b, c, and d) indicate significant differences (*p* < 0.05), whereas the same letters or no letters indicate no significant difference (*p* > 0.05).

## Data Availability

The original contributions of this study are in this article. For further inquiries, contact the corresponding authors.
